# Evidence of a conserved mammalian immunosuppression mechanism in *Lutzomyia longipalpis* upon infection with *Leishmania*

**DOI:** 10.3389/fimmu.2023.1162596

**Published:** 2023-11-02

**Authors:** Erich Loza Telleria, Bruno Tinoco-Nunes, David M. Forrest, Tatiana Di-Blasi, Tereza Leštinová, Kwang Poo Chang, Petr Volf, André Nóbrega Pitaluga, Yara Maria Traub-Csekö

**Affiliations:** ^1^ Department of Parasitology, Faculty of Science, Charles University, Prague, Czechia; ^2^ Laboratório de Biologia Molecular de Parasitas e Vetores, Instituto Oswaldo Cruz - Fiocruz, Rio de Janeiro, RJ, Brazil; ^3^ Department of Microbiology and Immunology, Chicago Medical School, Rosalind Franklin University of Medicine and Science, North Chicago, IL, United States

**Keywords:** sand fly, immunity, signaling pathway, protein-tyrosine phosphatase, SHP-2, vector-parasite interaction

## Abstract

**Introduction:**

Sand flies (Diptera: Phlebotominae) belonging to the *Lutzomyia* genus transmit *Leishmania infantum* parasites. To understand the complex interaction between the vector and the parasite, we have been investigating the sand fly immune responses during the *Leishmania* infection. Our previous studies showed that genes involved in the IMD, Toll, and Jak-STAT immunity pathways are regulated upon *Leishmania* and bacterial challenges. Nevertheless, the parasite can thrive in the vectors’ gut, indicating the existence of mechanisms capable of modulating the vector defenses, as was already seen in mammalian *Leishmania* infections.

**Methods, results, and discussion:**

In this study, we investigated the expression of *Lutzomyia longipalpis* genes involved in regulating the Toll pathway under parasitic infection. *Leishmania infantum* infection upregulated the expression of two *L. longipalpis* genes coding for the putative repressors cactus and protein tyrosine phosphatase SHP. These findings suggest that the parasite can modulate the vectors’ immune response. In mammalian infections, the *Leishmania* surface glycoprotein GP63 is one of the inducers of host immune depression, and one of the known effectors is SHP. In *L. longipalpis* we found a similar effect: a genetically modified strain of *Leishmania amazonensis* over-expressing the metalloprotease GP63 induced a higher expression of the sand fly SHP indicating that the *L. longipalpis* SHP and parasite GP63 increased expressions are connected. Immuno-stained microscopy of *L. longipalpis* LL5 embryonic cells cultured with *Leishmania* strains or parasite conditioned medium showed cells internalization of parasite GP63. A similar internalization of GP63 was observed in the sand fly gut tissue after feeding on parasites, parasite exosomes, or parasite conditioned medium, indicating that GP63 can travel through cells *in vitro* or *in vivo*. When the sand fly SHP gene was silenced by RNAi and females infected by *L. infantum*, parasite loads decreased in the early phase of infection as expected, although no significant differences were seen in late infections of the stomodeal valve.

**Conclusions:**

Our findings show the possible role of a pathway repressor involved in regulating the *L. longipalpis* immune response during *Leishmania* infections inside the insect. In addition, they point out a conserved immunosuppressive effect of GP63 between mammals and sand flies in the early stage of parasite infection.

## Introduction

Insects have several immune-related mechanisms to control commensal or potentially harmful microorganisms. Similar to mammals, their innate immunity involves cellular and humoral responses that are often tuned by different signaling pathways (1). The Toll and Janus kinase/signal transducer and activator of transcription (Jak-STAT) are among the most studied pathways in insects (2–4). They are activated by pathogen-associated molecular patterns (PAMPs) that bind to the host cell membrane receptors (5, 6). The Toll pathway activation requires first the binding PAMPs to the Spätzle ligand, and this complex subsequently binds to the Toll receptor (5, 7). The Jak-STAT pathway is activated when the unpaired (upd) family of ligands bind to the receptor domeless (dome) (2, 8). When these receptors are activated, a cascade of intracellular molecular events involving regulatory proteases and kinases results in the translocation of transcription factors to the nucleus. These events culminate with the transcription of genes associated with pathogen-killing effectors such as antimicrobial peptides (AMPs) and cytokines (2–4). Such activation can occur within minutes after an injury or microbial challenge.

Besides the pathway activation steps, other regulatory molecules can repress the downstream events of the immune response. These molecules can inhibit the pathway activity by blocking key molecular processes (9). For example, in *Drosophila*, the Toll pathway repressor cactus binds to nuclear factor kappa B (NF-κB) transcription factors preventing their translocation to the cell nucleus (10). For the Jak-STAT pathway, protein tyrosine phosphatases (PTPs) such as PTP16F (11) and sarcoma homology 2 (SH2)-domain-containing PTP (SHP) (12) repress Jak activity by dephosphorylation (13, 14). These regulatory mechanisms are crucial to prevent an uncontrolled response that may pose a high physiological cost (15).

This complex regulation of the innate immunity in insect vectors of public health importance gained significant attention (16–18). In *Aedes aegypti*, besides the response against bacteria and fungi, the Toll pathway was shown to be involved in the antiviral response against dengue virus (DENV) (19) and sindbis virus (20). Also, in *Anopheles* mosquitoes, Toll regulates the expression of the AMP gambicin against *Plasmodium* parasite (21). Moreover, the Jak-STAT pathway mediates resistance to DENV and ZIKV in the vector *A. aegypti*. Interestingly, the activation of this pathway through the knockdown of PIAS, one of the negative regulators, decreased the DENV infection (22, 23). The Jak-STAT pathway was also involved in responses to *Plasmodium* by *Anopheles aquasalis* (24, 25) and, in the case of *Anopheles gambiae*, with the transcriptional activation of nitric oxide synthase (NOS) (24).

In vectors of trypanosomatids such as tsetse flies, antimicrobial peptides were identified in the *Glossina morsitans* hemolymph after bacterial intrathoracic injection and after *Trypanosoma brucei brucei* oral infection (26). In triatomine kissing bugs, the successful infection of *Rhodnius prolixus* by *Trypanosoma cruzi* Dm 28c strain increased the expression of a defensin. In contrast, the *T. cruzi* Y strain did not complete the infection cycle in the insect and did not elicit AMPs expression (27). These examples show that trypanosome infections trigger their vectors’ immune response. Some components of the main pathways involved in the innate immune response have already been identified in sand flies. Toll, Jak-STAT and immune deficiency (IMD) gene expression in *L. longipalpis* Lulo and LL5 cell lines indicated that repressors of these pathways (cactus, caspar, and PIAS), as well as their transcription factors (dorsal, relish, and STAT), are differentially modulated depending on the microbial challenges (28–30).

In female *L. longipalpis*, activating the IMD pathway through the silencing of the repressor caspar favors *Leishmania mexicana* infection (31). Analogously, suppressing this pathway through the knockout of the pathway transcription factor Relish favored *Leishmania major* and bacteria growth in *Phlebotomus papatasi* (32). On the other hand, the Jak-STAT pathway regulators, such as PIAS and STAT showed no significant modulation when sand flies were infected by *L. infantum* (syn. *Leishmania infantum chagasi*). Moreover, the silencing of STAT caused the downregulation of an inducible NOS, and this effect was associated with increased *L. infantum* detection (30). These studies show that sand fly immunity affects the outcome of the parasite infection.

Although transcriptomic analysis indicated that *Leishmania* infection resulted in small overall transcriptional changes (33, 34), our previous results indicated that *Leishmania* experimental infection causes a broad spectrum of immune responses. For instance, the *L. mexicana* infection downregulated the expression of a *L. longipalpis* defensin gene in late phases of parasitic infection (35). On the other hand, when this sand fly was infected by *L. infantum*, there was an upregulation of three other AMPs in an earlier phase of infection (36). In addition, a gut-specific defensin was increased in *P. papatasi* depleted of gut bacteria and infected with *L. major* (37). Moreover, *L. i. chagasi* infection of *L. longipalpis* increased the expression of an activin-like gene that belongs to the Transforming Growth Factor-beta (TGF-β) family. Its suppression by RNAi caused a decline in parasite survival (38), suggesting that the parasite benefits from the sand fly expression of the TGF-β pathway.

There is evidence that *Leishmania* can modulate gene expression of immune regulatory molecules in *L. longipalpis* embryonic cells. Co-culture of *L. infantum* and LL5 cells caused the upregulation of PIAS and PTP61F genes associated with the Jak-STAT pathway’s repression (30). Lessons learned from *Leishmania*-vertebrate host studies show that parasite virulence factors can be responsible for modulating the host immune response. For example, glycosylinositol phospholipids (GIPLs), lipophosphoglycans (LPGs), proteophosphoglycans (PPGs), and the glycoprotein GP63 are among the *Leishmania* molecules involved in the host cell invasion and maintenance of chronic infection (39, 40). In fact, *Leishmania* GP63, a metalloprotease, activates the murine SHP-1, which represses the Jak-STAT (41) and Toll (42) pathways.

We hypothesized that, similar to the mammalian infection model, a sand fly immunity pathway repressor could be the target of a *Leishmania* virulence factor such as GP63. To address this possibility, we focused on Toll-related pathway genes. We selected two *L. longipalpis* genes, cactus and SHP-2, coding for repressors involved in the Toll pathway and explored their expression under *Leishmania* infection conditions. We used RNAi-mediated gene silencing to test the role of SHP-2. In addition, we tested whether the parasite GP63 interfered with the sand fly immunity and whether it could be internalized by *L. longipalpis* cells. In the present study, we report on the sand fly expression of SHP-2 and its possible connection with a parasite virulence factor.

## Materials and methods

### Gene identification

*L. longipalpis* partial coding sequences for the Toll pathway repressor cactus were previously identified (28). Partial coding sequences for an additional repressor associated with the Toll pathway SHP-2 were identified from the National Center for Biotechnology Information (NCBI) (Bethesda, MD, USA) dbESTs database (43) and the *L. longipalpis* genome available at VectorBase database (44). Similar sequences from *Drosophila*, *Aedes*, and *Culex* were used as a query on blastx and tblastx search against these databases. The obtained sand fly sequences were reversely checked using the same programs against the NCBI non-redundant (nr) protein sequences database (45). The OrthoMCL search tool (46) was used for homology confirmation. The InterPro (47) and the NCBI Conserved Domain Database (48) were used to identify conserved domains to support the sequences’ identity.

Similarities of *L. longipalpis* cactus and SHP-2 amino acid sequences shared with other insects were assessed by MUSCLE multiple sequence alignments (49) built-in Geneious 7.1.9 software (Biomatters, New Zealand). Cladograms were created using MEGA-X software (50) with the maximum likelihood method. According to the lowest Bayesian Information Criterion (BIC) score, the best substitution model was estimated using MEGA X software. Cactus analysis was done using the Jones-Taylor-Thorton (51), while SHP-2 analysis was done using the Le-Gascuel (52) substitution models. Evolutionary rates among sites were modeled using a Gamma distribution with invariable sites for the phylogeny analyses. A bootstrap of 400 replications was used to model evolutionary rate differences among sites.

### Sand fly and parasite cell cultures

*L. longipalpis* embryonic LL5 cells (53) were grown in L-15 medium (Sigma, USA) supplemented with 10% heat-inactivated fetal bovine serum (HI-FBS) (Cultilab, Brazil), 10% tryptose phosphate broth (TPB) (Sigma), and 1% antibiotics (penicillin 100 U/mL and streptomycin 100 mg/mL) (Sigma).

*Leishmania amazonensis* (MPRO/BR/72/M1845/LV78) transfected with P6.5/1.9R and P6.5/1.9 for knockdown and over-expression of GP63 metalloprotease, respectively (54) were maintained at 25°C in medium 199 (Thermo Fisher Scientific, USA), pH 7.0, supplemented with 10% HI-FBS under the selective pressure of tunicamycin (10 µg/mL) (Sigma). Wild-type *L. amazonensis* (BMVirWT) promastigotes were maintained in the same medium without tunicamycin.

*L. infantum* (syn. *L. i. chagasi*) (MHOM/BR/1974/PP75) obtained from the *Leishmania* collection of Instituto Oswaldo Cruz (CLIOC - Fiocruz/IOC, Brazil) was maintained at 25°C in medium 199, pH 7.0, supplemented with 10% HI-FBS. Promastigotes of *L*. (Viannia) *braziliensis* (MHOM/BR/75/M2904) were grown at 26°C in Schneider’s *Drosophila* medium (Sigma) supplemented with 10% heat-inactivated fetal bovine serum (FBS), 1% glutamine, 100 U/ml penicillin, and 100 mg/ml streptomycin (55).

### *Leishmania* exosome purification

*L. infantum* culture was initiated with 10^6^ parasites/mL and grown for three days. Parasites were pelleted by centrifugation at 1,500 x g for 10 min at 4 °C and washed three times with non-supplemented medium 199. Parasites were resuspended in fresh medium 199 supplemented with TPB instead of FBS and grown for 24 h at 26 °C.

For exosome recovery, cultures were submitted to differential centrifugation at 4 °C as follows: 300 x g for 10 min to remove live parasites, 2,000 x g for 10 min to remove dead parasites, 10,000 x g for 30 min to remove cellular debris, and finally, ultracentrifugation at 100,000 x g for 60 min to pellet exosomes. Exosome pellets were resuspended in ice-cold phosphate buffered saline (PBS, pH7.4), washed, and submitted to additional ultracentrifugation (56). The final exosome pellets were resuspended in PBS and used in incubation with LL5 cells or sand fly feeding procedures. Protein quantification of exosomes was performed using the Pierce 600nm colorimetric assay. To note, in our previous proteomic analysis of *L. infantum* exosomes prepared using this protocol (56), 50 out of 53 exosome markers that have homologues to the ExoCarta database were revealed (57).

### Incubation of LL5 cells with *Leishmania*

LL5 cells were cultured in a 24-well flat-bottom plate at a density of 5 x 10^5^/well in 500 μL of L-15 media supplemented with 10% TPB and 1% antibiotics (penicillin 100 U/mL and streptomycin 100 mg/mL) (Sigma) for 24 h at 30 °C to allow cell attachment to the bottom of the wells. Cells were washed 3x with fresh L-15 medium, L-15 medium containing *Leishmania* parasites at a 10 to 1 parasite/LL5 cell ratio was added to the wells, and plates were incubated for one hour at 30 °C. LL5 cells cultured under the same conditions without parasites were used as control. Plates were subsequently used for visualization of *Leishmania* GP63 by immunofluorescent microscopy.

### Incubation of LL5 cells with *Leishmania* conditioned medium

*Leishmania* conditioned medium was obtained from 7 days cultures after clearance by centrifugation at 2,000 x g for 10 min. LL5 cells were cultured in a 24-well flat-bottom plate as described above. After the cell wash, *Leishmania* conditioned medium was added to the LL5 cells-containing wells and incubated for one hour at 30 °C. Plates were subsequently used for *Leishmania* GP63 immunofluorescent microscopy. Fresh 199 medium was used instead of *Leishmania* conditioned medium as the control.

### *Lutzomyia longipalpis* colony

Sand fly colony was previously established from *L. longipalpis* originally collected in Jacobina, BA, Brazil, and kept at temperatures between 24-28 °C and 70-80% relative humidity under standard insectary conditions (58). For daily colony maintenance, adult insects were allowed to feed on 50-70% sucrose solution *ad libitum*, and females were blood-fed once a week on anesthetized hamsters or mice. The use of all animals was reviewed and approved by the Committee on the Ethics for the use of Animals in the Institute Oswaldo Cruz (CEUA-IOC) under permission No. CEUA/IOC-005/2019 and the Committee on the Ethics of Laboratory Experiments of Charles University under permission No. MSMT-8604/2019-6.

### *Lutzomyia longipalpis* artificial feeding with *Leishmania* exosomes

Three to 6 days old female sand flies were fed through a chickskin membrane, using a Hemotek system, with heat-inactivated rabbit blood (under permission No. CEUA/IOC-005/2019) seeded with *Leishmania* exosomes at 40 μg/mL. Sand flies fed on blood without exosomes were used as control groups. Only fully engorged females from both experimental and control groups were used.

### *Lutzomyia longipalpis* artificial infection with *Leishmania*

Sand flies were artificially fed as described on heat-inactivated rabbit blood seeded with *L. infantum* or *L. amazonensis* (10^7^ parasites/mL of blood) obtained from exponential growth culture. Sand flies fed on blood without parasites were used as control groups. Fully engorged females were separated and collected at different times post-infection for RNA extraction and microscopy analysis. A 70% sucrose meal was offered *ad libitum* after blood feeding. Refer to figure legends for more details.

### RNA extraction and cDNA synthesis

RNA was extracted from pools of 10 females by homogenization per manufacturer’s instructions in TRIzol reagent (Thermo Fisher Scientific) and stored at -80 °C until used. RNase-free DNase I (Thermo Fisher Scientific) digestion step was added to remove DNA. The cDNA synthesis was done using SuperScript III Reverse Transcriptase (Thermo Fisher Scientific) using up to 1 μg of total RNA as template following the manufacturer’s instructions.

### RNAi-mediated gene silencing

The *L. longipalpis* SHP-2 gene was submitted to RNAi-mediated gene silencing experiments. Gene-specific primers (dsSHP2-F and dsSHP2-R) coupled to T7 promotor sequence (**Table 1**) were designed to amplify DNA templates for dsRNA synthesis reactions. A DNA template was amplified from p-GEM-T Easy plasmid (Promega, USA) with dsLacZ-F and dsLacZ-R primers (**Table 1**) to produce a control dsRNA. The templates were amplified by a touchdown PCR under the following cycling conditions: 95 °C for 3 min; 16 cycles of 95 °C for 45 sec, 68 to 50 °C (progressively decreasing 1°C per cycle) for 45 sec, and 72°C for 45 sec; 26 cycles of 95 °C for 45 sec, 50 °C for 45 sec, and 72 °C for 45 sec; 72 °C for 3 min.

**Table 1:** Oligonucleotides.

Reference	Name	Sequence
Present study	dsSHP2-F	taatacgactcactatagggagaCAGACACAGGAATGGGGA #
	dsSHP2-R	taatacgactcactatagggagaGGCGTAGTAGACAAACTGT #
(59)	dsLacZ-F	taatacgactcactatagggagaTATCCGCTCACAATTCCACA #
	dsLacZ-R	taatacgactcactatagggagaGAGTCAGTGAGCGAGGAAGC #
(28)	Cactus-F	CTAATCCGAATGAATCCCTACCC
	Cactus-R	GACCCACGATCACGGCTAGA
GenBank KP030756	SHP2b-F	GCATGCCGAACACGATAATT
	SHP2b-R	CTTATTCTTACGCCGCTCGT
(60)	LeishActin-F	GTCGTCGATAAAGCCGAAGGTGGTT
	LeishActin-R	TTGGGCCAGACTCGTCGTACTCGCT
(61)	RP49-F	GACCGATATGCCAAGCTAAAGCA
	RP49-R	GGGGAGCATGTGGCGTGTCTT

#Lowercase nucleotides indicate T7 promoter sequence.

SHP-2 and LacZ templates were used in dsRNA synthesis reaction by MEGAscript RNAi kit (Thermo Fisher Scientific) following the manufacturer’s instructions. The produced dsRNA was concentrated to 4.5 μg/μL, and 32.2 nL were microinjected intrathoracically into *L. longipalpis* females using Nanoject II microinjector (Drummond, USA) (62). The dsRNA injected flies were kept under colony maintenance conditions with sucrose feeding and used on the following day for *Leishmania* infection experiments. Refer to figure legends for more details.

### Relative gene expression

The relative expression of selected genes was assessed by qPCR using *L. longipalpis* cDNA samples from various samples as described under different experimental settings. Gene-specific primer sets used for qPCR are listed in **Table 1**. Cycling conditions followed manufacturer’s standard procedures using SYBR Green PCR Master Mix in a 7500 Real-Time PCR System (Applied Biosystems, USA). Specific gene expression was calculated relative to a ribosomal protein (RP49) reference gene (28) and expressed in fold-change values compared to a control group defined according to each experimental design. Refer to figure legends for more details.

### Immunofluorescence detection of *Leishmania* GP63

Midguts from artificially blood-fed *L. longipalpis* females were dissected in PBS at 2 h and 24 h post-feeding, and the ingested bloodmeal content was removed. Midguts were incubated in 4% paraformaldehyde (PFA) for 30 min, transferred to 0.5% triton X-100 for 20 min, and to 3% BSA for 40 min. The midguts were then washed 3x in PBS for 10 min and incubated with primary anti-GP63 antibody (1:5,000 dilution) in a 1% BSA, 0.25% triton X-100 solution overnight at 4 °C. Midguts were subsequently washed 3x in PBS for 10 min and incubated with goat anti-rabbit IgG (H+L) Cross-Adsorbed Secondary Antibody, Alexa Fluor™ 546 (Thermo Fisher Scientific) diluted in 1% BSA, 0.25% triton X-100 solution for 1 hour. After additional 2x wash steps in PBS for 10 min samples were incubated with DAPI for 10 min followed by a final 1x wash in PBS for 10 min. Midguts were transferred to glass slides for subsequent confocal microscope analysis using a Leica DMi8 confocal microscope (Leica, Germany).

### *Leishmania* development in sand fly guts

Eight days post-infection, *Leishmania* loads, location, and development were assessed by light microscopy in *L. longipalpis* females silenced for SHP-2 and LacZ. A minimum of 20 sand fly guts from each group were examined. Sand flies were anesthetized on ice and transferred to a saline solution (0.9% NaCl) for dissection of the guts. Parasite loads were estimated under 40x magnification objective lens and classified as low (below 100 parasites), medium (between 100 and 1,000 parasites), or heavy infection (above 1,000 parasites) (63). Simultaneously, localization of parasites in various portions of the sand fly midgut was recorded (63, 64).

To assess the parasite developmental stages, sand fly gut smears were prepared on glass slides and Giemsa-stained. The glass slides were inspected using an Olympus BX51 microscope (Olympus, Japan) under a 100x magnification objective lens. Images of 400 randomly selected *Leishmania* promastigotes were captured from two independent dsRNA injected sand fly groups. The width and length of cells and their flagellum were measured using the microscope scale plugin in ImageJ 1.52a software (65). Different categories of parasites were defined as elongated nectomonads (body length ≥ 14 μm), procyclic promastigotes (body length < 14 μm and flagellar length ≤ body length), metacyclic promastigotes (body length < 14 μm and flagellar length ≥ 2x body length), and leptomonads (remaining parasites) (64, 66).

### Statistical analysis

Ordinary two-way ANOVA with Sidak’s correction for multiple comparisons test was used to calculate significant differences in gene expression levels obtained by qPCR. This same method was applied to assess significant differences in infection estimation, localization, and parasite stages from data obtained by light microscopy observations. The statistical analysis was carried in GraphPad Prism software (version 6.07) (GraphPad Software Inc., USA).

## Results

### *Lutzomyia longipalpis* immunity gene sequences

Cactus gene sequences were previously identified (28). The additional *L. longipalpis* coding sequences of SHP-2 (GenBank KP030756) were deposited in the NCBI GenBank database. We analyzed sequences of the two Toll-related repressors selected in the present work. The deduced amino acid sequence of cactus contains a domain of six ankyrin repeats present in the inhibitor of NF-κB (I-κB) family (67, 68). The phylogenetic analysis showed that the *L. longipalpis* sequence formed a group with a *P. papatasi* sequence and was included in a larger group of I-κB sequences from dipterans such as *Drosophila*, *Bactrocera*, and *Rhagoletis* flies. The vertebrate I-κB formed an outgroup composed of I-κBα, I-κBβ, and I-κBε sequences (**Figure S1**).

The deduced amino acid sequence of SHP-2 (GenBank AKU77025) contains the catalytic domain of tyrosine-protein phosphatase non-receptor type 11 and type 6 (PTPn11 and PTPn6) (CDD cd14544), which contains the sarcoma homology 2 (SH2) (IPR000980) and tyrosine-specific protein phosphatase PTPase (IPR000242) domains. The PTPn11 and PTPn6 are also known as SHP-2 and SHP-1, respectively. The phylogenetic analysis showed that the *L. longipalpis* SHP-2 amino acid sequence formed a separate branch from the mosquito SHP-2/PTPn11 sequences. It is included in the larger group of dipterans together with *Drosophila*, *Musca*, and *Stomoxys* corkscrew sequences. The vertebrate PTPn6 sequences formed an outgroup (**Figure S2**).

### Expression of Toll pathway regulators during sand fly *Leishmania* infection

**Figure 1:**
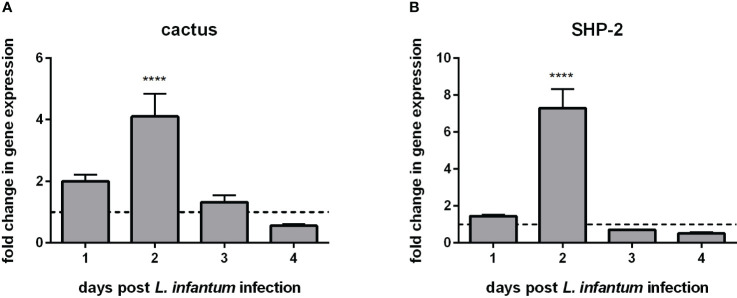
Gene expression of Toll-related repressors in *L. longipalpis* infected with *L. infantum*: **(A)** expression of cactus and **(B)** expression of SHP-2. Relative gene expression was calculated compared to the endogenous reference gene RP49 and expressed as fold change (y-axis) compared to the non-infected control group (horizontal dotted line). Samples of experimental and control groups were collected in pools of 10 sand flies on days 1, 2, 3, and 4 post-*Leishmania* infection (x-axis). Vertical bars represent the mean with standard error (SEM) of 3 biological replicates. The differences were significant, as calculated using two-way ANOVA (**** p < 0.0001).

We chose an experimental setting representing a potential challenge to the sand fly’s immunity and investigated the gene expression of the two Toll-related repressors, cactus and SHP-2. Considering that in the mammalian model *Leishmania* infection of macrophages modulates their immune response (41, 42), we tested and proved the hypothesis that the sand fly repressors cactus and SHP-2 were also modulated under the same infectious conditions. Our results showed that the transcription of cactus and SHP-2 increased on the second day post-infection (**Figures 1A, B**).

### Expression of SHP-2 in *L. longipalpis* infected with GP63-over and under-expressing *Leishmania*

**Figure 2:**
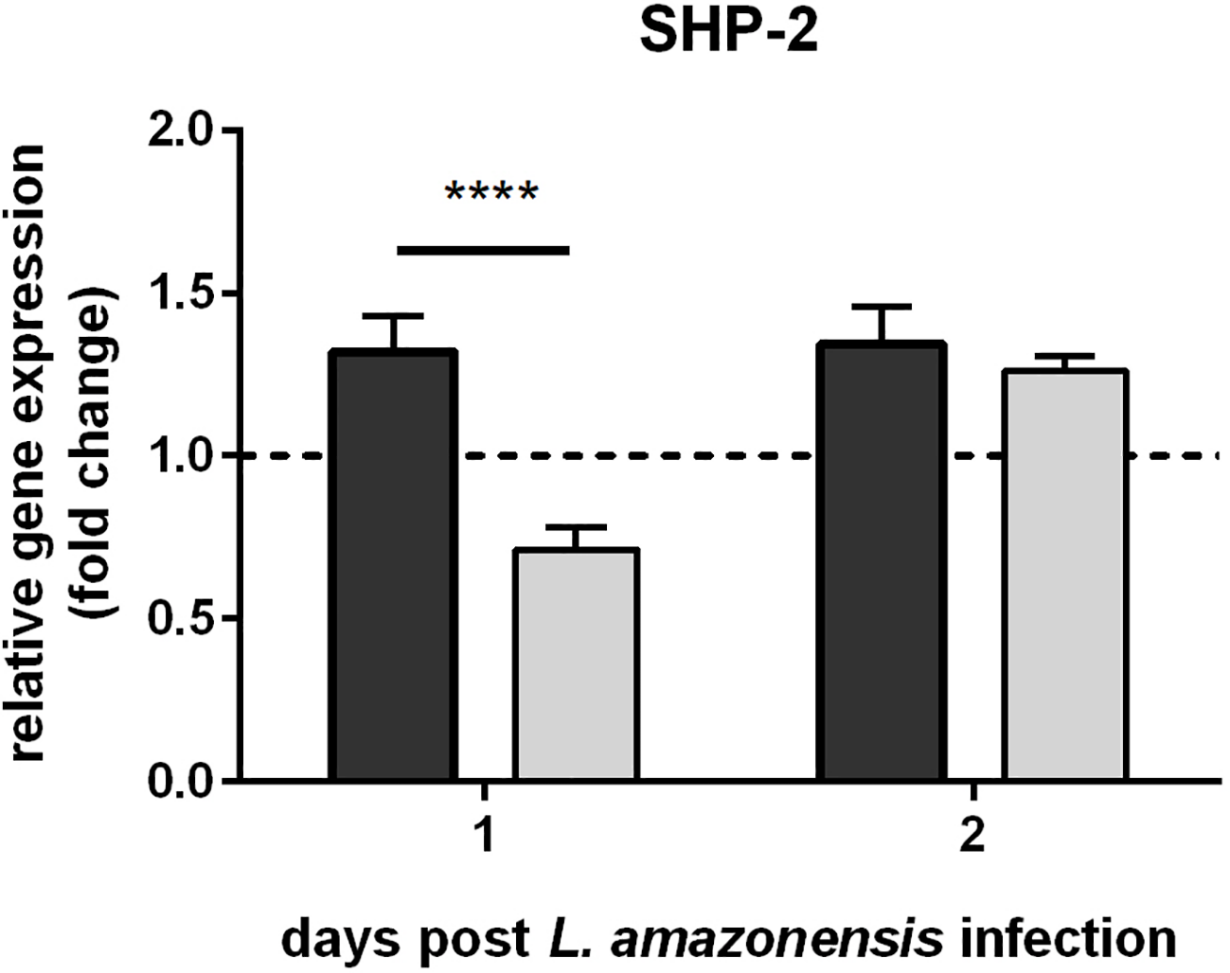
SHP-2 gene expression in *L. longipalpis* infected with *L. amazonensis* up- and down-regulated expression of GP63: SHP-2 relative gene expression was calculated with reference to the constitutively expressed ribosomal gene RP49 and expressed as fold change (y-axis) in comparison to the control group infected with wild type *L. amazonensis* strain (horizontal dotted line). Dark and light grey bars represent SHP-2 expression in sand flies infected with GP63-overexpressing and -underexpressing *L. amazonensis*, respectively. RNAs were isolated from a pooled ssample of 10 sand flies for each group on days 1 and 2 post-*Leishmania* infection (x-axis). The mean with standard error (SEM) shown was calculated from 3 biological replicates in each group. Statistical significance was calculated using two-way ANOVA (**** p < 0.0001).

The gene expression analysis of *L. infantum*-infected sand flies indicated that such infection increased sand fly immunity repressors. In macrophages, the *Leishmania* GP63 metalloprotease is one of the molecules responsible for suppressing the mammalian host immune response (41, 42). We showed a similar action of this protease in sand fly females by infecting them with strains of *L. amazonensis* showing up- and down-regulated expression of GP63. SHP-2 expression was elevated in the sand flies infected with overexpressing GP63 parasites in relation to infections with *Leishmania* with down-regulated GP63 (**Figure 2**).

### Uptake of GP63 from *Leishmania* by sand fly cells *in vitro*

**Figure 3:**
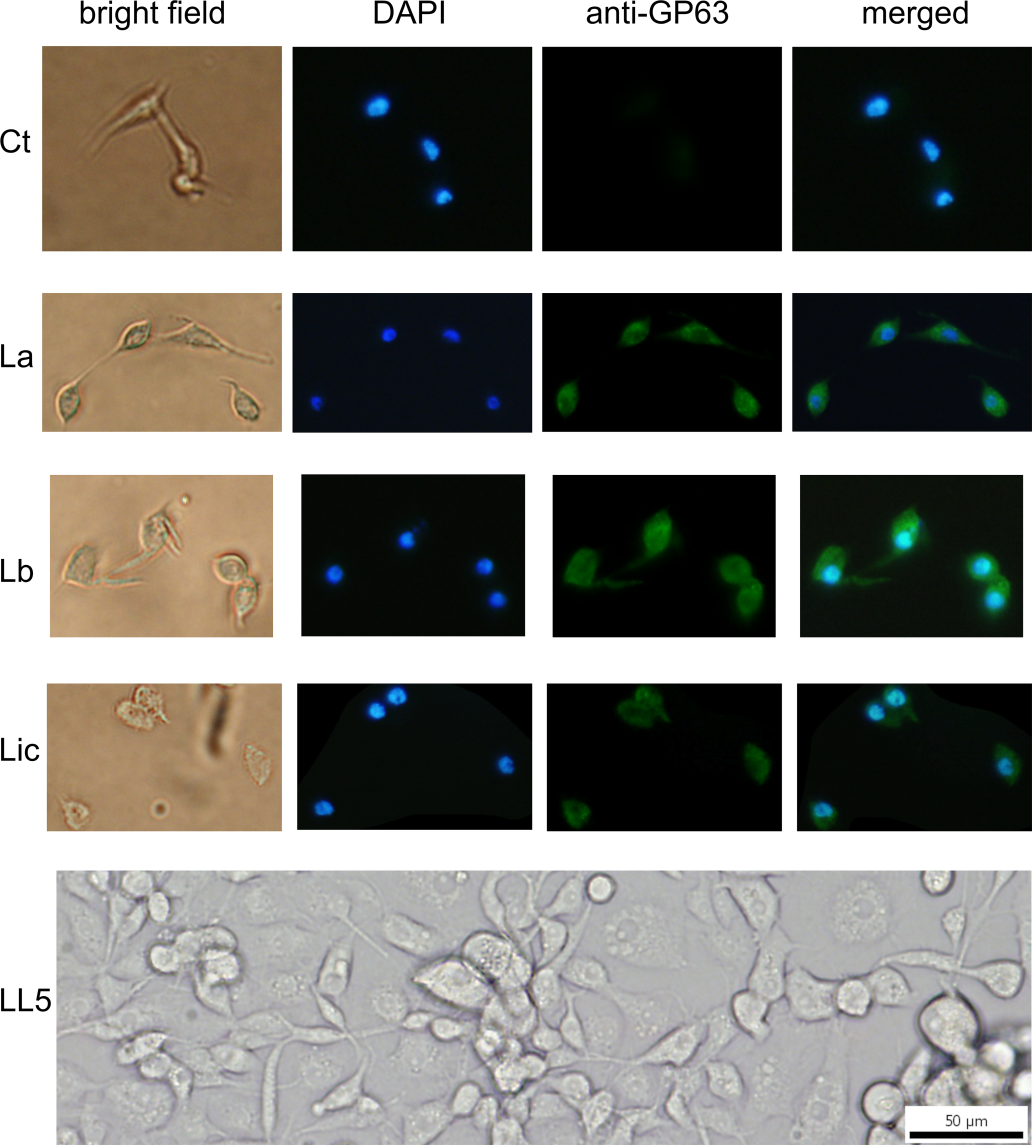
GP63 detection in LL5 cells after co-culture with *Leishmania* parasites: *L. longipalpis* LL5 embryonic cells were co-cultured for one hour at 30 °C with each of the three different *Leishmania* species separately. These cells were submitted to fluorescent microscopy after incubation with DAPI and anti-GP63 FITC-antibody. Ctr – LL5 control culture without *Leishmania*; La – *L. amazonensis*; Lb – *L. braziliensis*; Li – *L. infantum*. Bright field, DAPI (blue), anti-GP63 antibody (green), and merged images were aligned vertically. LL5 – control cell culture.

The uptake of GP63 was demonstrated by co-culture of *L. longipalpis* LL5 cells with three different *Leishmania* spp., i.e. *L. amazonensis*, *L. braziliensis*, and *L. infantum*. By immunofluorescence microscopy using an anti-GP63 antibody, the association of GP63 with LL5 cells was detected only when they were co-cultured for one hour with any of the three *Leishmania* species (La, Lb, and Li) used, but not with medium alone as control (Ctr) (**Figure 3**).

### Association of GP63 with LL5 cells after incubation with *Leishmania*conditioned medium

**Figure 4:**
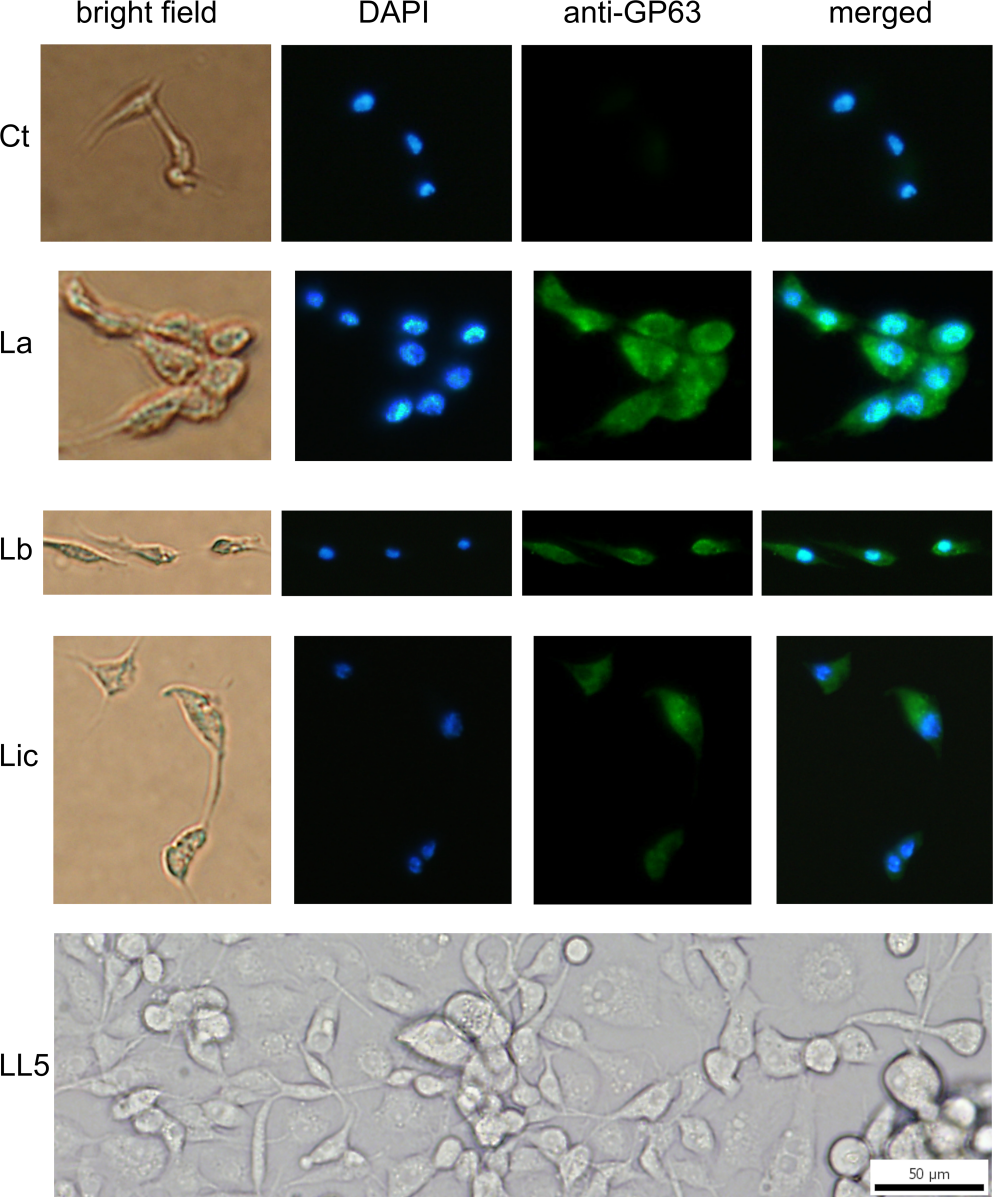
GP63 detection in LL5 cells after incubation with *Leishmania* conditioned medium: *L. longipalpis* LL5 embryonic cells were incubated for one hour at 30 °C with conditioned medium from each of the three different *Leishmania* species. Fluorescent microscopy with DAPI and anti-GP63 antibody was as described. Ctr – LL5 control without *Leishmania* conditioned medium; La – *L. amazonensis*; Lb – *L. braziliensis*; Li – *L. infantum*. Bright field, DAPI (blue), anti-GP63 antibody (green), and merged images were aligned vertically. LL5 – control cell culture.

Association of *Leishmania* GP63 with LL5 cells was also found under exactly the same experimental conditions as described for **Figure 3**, except that *Leishmania* conditioned or spent media were used instead of *Leishmania* cells (**Figure 4**). This observation is consistent with the previous finding that *Leishmania* promastigotes shed GP63 into their culture medium (56).

### Localization of GP63 in *L. longipalpis* gut after feeding on blood containing *L. infantum* exosomes, parasites or conditioned medium

**Figure 5:**
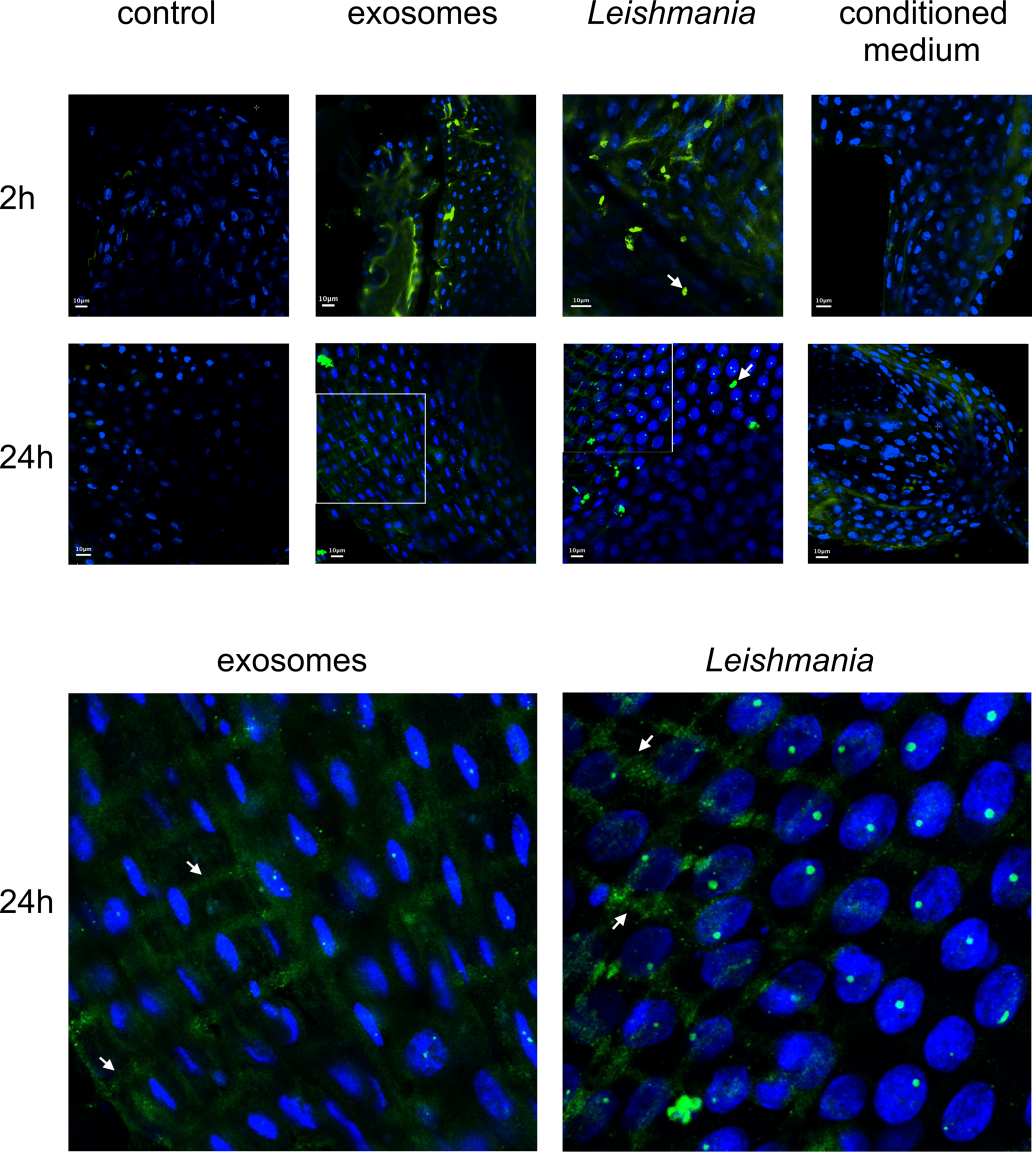
Confocal microscope images of midguts from sand flies fed on blood containing *L. infantum* exosomes, parasites, or conditioned medium: Midguts were dissected at 2 h or 24 h after artificial blood-feeding (top section) with blood only (control), blood seeded with *L. infantum* exosomes (exosomes), *L. infantum* parasites (*Leishmania*), and *L. infantum* conditioned medium (conditioned medium). Parasites are indicated by white arrows (top section). Criss-cross midgut muscle and small sand fly vesicular structures are indicated by white arrows in the 4.25 times zoomed images from 24 h guts (exosomes and *Leishmania*) (bottom section). Green = FITC-anti-GP63 antibody, Blue = DAPI.

Our findings of *Leishmania* GP63’s association with LL5 cells *in vitro* led us to examine this *in vivo* in the midgut cells of female sand flies fed with three different blood meal mixtures: a) blood containing *L. infantum* exosomes; b) blood containing *L. infantum* parasites; c) blood containing *L. infantum* conditioned medium. The artificial feeding with blood containing exosomes or *Leishmania* showed that GP63 was detected in midguts dissected 2 h after feeding and largely cleared from the gut lumen by 24 h post-feeding. Although less intense, GP63 was also detected in the guts of sand flies that ingested the conditioned medium. Additionally, GP63 can be detected as traversing the midgut epithelium, colocalizing with the midgut muscular fibers and small vesicular structures. A similar GP63 localization was also observed in midguts exposed only to parasites (**Figure 5**).

### Silencing of SHP-2 in sand flies reduces their *Leishmania* infection

**Figure 6:**
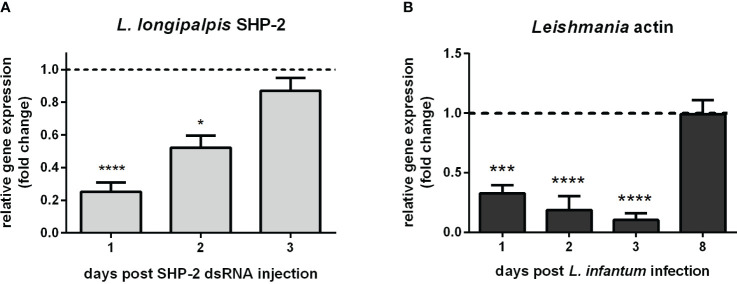
*L. longipalpis* SHP-2 and *Leishmania* actin gene expression in SHP-2-silenced and *L. infantum*-infected females: **(A)** SHP-2 gene expression in SHP-2 dsRNA injected sand flies. Relative gene expression was calculated compared to the endogenous reference gene RP49 and expressed as fold change (y-axis) compared to the control group injected with LacZ dsRNA (horizontal dotted line); **(B)** parasite detection through the relative expression of *Leishmania* actin gene in SHP-2 silenced and *L. infantum*-infected sand flies. Relative gene expression was calculated with reference to the endogenous reference gene RP49 and expressed as fold change (y-axis) by comparison to the control group injected with LacZ dsRNA and infected with *Leishmania* (horizontal dotted line). Samples of experimental and control groups were collected in pools of 10 whole sand flies on day 1, 2, 3, and 8 post*-*parasite infection (x-axis). Vertical bars represent the mean with standard error (SEM) of 3 biological replicates. Significant differences were calculated using two-way ANOVA (* p< 0.05; *** p< 0.001; **** p < 0.0001).

The foregoing data presented led us to suppress the expression of SHP-2 by RNAi-mediated gene silencing in sand flies for testing their susceptibility to *Leishmania* infection. Injection of SHP-2 dsRNA successfully reduced the SHP-2 gene expression most significantly on day 1 followed by its gradual recovery in the next 2 days (**Figure 6A**). *L. infantum* infection of the SHP-2 dsRNA injected group was increasingly suppressed for 3 days and did not return to the level of the control group until day 8 (**Figure 6B**).

### *Leishmania* development in sand flies after transient RNAi-silencing of their SHP-2

**Figure 7:**
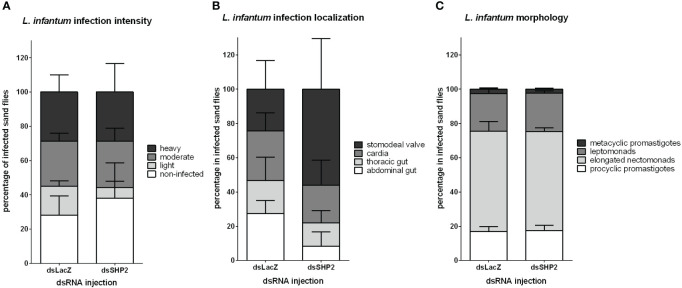
*L. infantum* development in SHP-2-silenced *L. longipalpis*: *L. infantum* infection intensity, location and developmental stages (8 days post-infection) in dsRNA-injected sand flies. **(A)** The y-axis represents the percentage of all individually inspected insects (minimum of 20 sand flies in each dsRNA injected group). Bars from white to shades of grey indicate infection intensity or parasite loads: non-infected (white), low or light infection (light grey), moderate or medium infection (mid grey), and heavy infection (dark grey). **(B)** The y-axis represents the percentage of infected insects used in infection progress evaluation in the gut. Bars from white to dark grey indicate *Leishmania* location in the sand fly gut: parasites reached the stomodeal valve (dark grey), cardia (mid grey), thoracic gut (light grey) or stayed in the abdominal gut (white). **(C)** Bars from white to dark grey indicate parasite developmental stages: metacyclic promastigote (dark grey); leptomonad (mid grey); elongated nectomonad (light grey); procyclic promastigote (white). The x-axis represents dsRNA-injected groups. No significant differences were found between the experimental and control groups (two-way ANOVA).

The parasite detection by qPCR indicated that the SHP-2 gene silencing decreased *Leishmania* numbers in the early phase of infection. The assessed amount of parasite was not significantly changed in the SHP-2 silenced group later in the parasitic infection. Nevertheless, the initial parasite reduction could cause changes in the gut colonization and parasite differentiation progress. We estimated the infection load, assessed the parasite localization in *L. longipalpis* gut, and evaluated the *Leishmania* morphology 8 days post-infection. At this time point, as infection advances and debris of blood digestion are excreted, parasites should be detectable in several parts of the sand fly gut, and the infective forms of the parasite become abundant. No significant differences were detected in infection levels in SHP-2-dsRNA injected sand flies compared to the LacZ-dsRNA injected control group (**Figure 7A**). There was a quite variable, thus non-significant detection of parasites in the stomodeal valve in SHP-2-dsRNA injected groups (**Figure 7B**). We also evaluated the parasite morphology in Giemsa-stained gut smears, and no significant differences were observed in parasite developmental forms between the dsRNA-injected groups (**Figure 7C**).

## Discussion

The complex balance of the sand fly immune response toward pathogens is a key process for vectorial transmission outcome. We chose to investigate key regulatory genes involved in inhibiting immune pathways upon insect vector infection by *Leishmania*, in search of mechanisms reminiscent of what is seen in the parasite**-**mammalian host interactions.

*L. longipalpis* gene sequence homologous to cactus, a Toll pathway repressor (69), was previously identified (28, 70, 71). Cactus is the invertebrate homolog of the vertebrate I-κB family that includes I-κBα and I-κBβ. Our phylogenetic analysis showed that the *L. longipalpis* cactus peptide sequence was closely related to *Drosophila* sequences, in agreement with the I-κB evolutionary analysis by (72). The analyses of signature domains and similarities shared with other dipteran sequences increased supporting information for the previously identified sequences.

Another Toll-related regulator is SHP-2 (73), a protein tyrosine phosphatase, which is involved in PTP-mediated phosphorylation, an important cellular regulatory mechanism. Specifically, SHPs are key modulators of important immunity pathways, including Toll in mammals (42, 74). The *L. longipalpis* SHP-2 translated sequence contains the PTPn11 and PTPn6 signature domains of the SHP protein subfamily in vertebrates and invertebrates (75). Their signature domains comprise two SH2 domains interacting with phosphotyrosine docking sites and a PTP catalytic domain. The *L. longipalpis* SHP-2 sequence shares similarities with mosquito PTPn11 and fruit fly corkscrew sequences that are homologous to the mammalian SHP (76–78).

Like many other insects, the sand fly immune response can be activated to fight infections or balance the natural microbiota (79). The role of cactus in regulating the downstream expression of AMPs in *L. longipalpis* was first investigated in the LL5 cells. The RNAi-mediated gene silencing in non-challenged cells resulted in the upregulation of cecropin and defensin 2 (28). This finding reflected an association of the repressor suppression with the upregulation of effector molecules related to the Toll pathway.

The sand fly immune responses interact with and respond differently to commensal and harmful microbes. For instance, sand fly females can produce an anti-bacterial response in the hemolymph after experimental injections with Gram-positive or -negative bacteria (80, 81). In addition, the gene expression of AMPs in *L. longipalpis* increased when females were experimentally fed on sucrose meal seeded with bacteria (35) or when bacteria loads in the gut increased after feeding on the regular sucrose meal (36). In *Leishmania*-infected sand flies, defensin 1 gene expression was reduced after *L. mexicana* infection (35), while attacin, cecropin, and defensin 2 expression was increased after *L. infantum* infection (36). In these last two studies, the differential expression was assessed in whole-body samples, indicating several tissues’ immune responses. On the other hand, a transcriptome analysis of the *L. longipalpis* midguts showed that immunity genes are not among the most differentially expressed upon *L. infantum* infection, especially in a late phase of infection (33). Therefore, the systemic response may have a major role in the sand fly immunity, while the gut response is stable regardless of the parasitic infection.

Another possibility is that the sand fly tolerates *Leishmania* infection to a certain point due to the oral infection route of infection, which avoids inner cell responses and other immune repertoire like cellular immunity from hemocytes in the hemolymph. Interestingly, in *Anopheles* mosquitoes, there is a threshold of the number of *Plasmodium berghei* parasites that can infect the vector without eliciting a complement-like response in the hemocoel (82).

We investigated here the possibility of the parasitic infection modulating the Toll pathway repressors in *L. longipalpis*. We selected cactus and SHP-2 that are involved in regulating the Toll pathway. These two repressors were upregulated two days post *L. infantum* infection, indicating that this pathway is repressed in the early phase of the parasite infection cycle, possibly reflecting suppression of the Toll pathway. The *Leishmania* procyclic promastigotes might cause this effect on the initial days of infection. It is unlikely that such upregulation happened due to close contact of parasites with the sand fly gut epithelium. Before the end of blood digestion, the sand fly peritrophic matrix is not completely degraded, and parasites are enclosed in the food bolus (83). Therefore, a parasite-secreted molecule is envisioned to mediate the upregulation of cactus and SHP-2, thus reducing the vector’s immune response. Another interesting example of parasite-induced changes to the host occurs with *P. falciparum* parasites expressing the Pfs47 gene that codes for a 6-cysteine protein family protein and prevents the activation of several caspases. Consequently, it inhibits JNK-mediated apoptosis (84, 85).

Among several molecules secreted by the parasite, GP63 is a well-studied molecule involved in the modulation of the vertebrate host’s immune response. Indeed, the *Leishmania* GP63 was implicated in activating the murine macrophage SHP-1 leading to a down-modulation of the Toll pathway. The SHP family has a single homolog in invertebrates (86); therefore, it is possible that the sand fly SHP-2 has a role similar to the mammalian SHP-1. Our results from *Leishmania*-sand fly data here presented showed that the parasite GP63 induces insect gene expression. Interestingly, Hassani and Olivier (87) showed that GP63 carried by exosomes increased the production of a series of proteins from uninfected macrophages, including SHP-1 (87). Therefore, our finding that this parasite metalloprotease caused a gene upregulation in the sand fly is quite significant.

Moreover, our results indicate that there is a common strategy shared by *L. infantum* and *L. amazonensis*, which appears beneficial to the parasite for colonization in the insect gut. In the current study, *L. amazonensis* over-expressing GP63 resulted in an increased number of infected *L. longipalpis* females in the early phase of infection. In contrast, the strain under-expressing GP63 resulted in fewer infected sand flies (88). Previous quantification of GP63 in these strains, measured by flow cytometry, revealed a more than 16-fold difference between the over-expressing and under-expressing strains (54). These results indicate that the GP63 expression and the SHP-2 upregulation are connected, favoring the parasite infection of the vector similar to that shown in the macrophages (41, 42).

The LL5 embryonic cells co-cultured with different *Leishmania* species and *Leishmania* conditioned medium showed that parasite produced GP63 for internalization by these sand fly cells. Therefore, our results showed a similar event between the metalloprotease and the insect cells *in vivo*. The *Leishmania* conditioned medium contains a complex mixture of secreted molecules, of which several are known virulent factors, including GP63 (89–92). We also detected the association of parasite metalloprotease with the LL5 cells, independent of a direct contact between live parasites and sand fly cells.

It is possible that during the *Leishmania* developmental cycle within the sand fly, GP63 is made available to modulate the sand fly epithelial gut cells response, triggering the suppression of insect immunity. We experimentally fed *L. longipalpis* females with different mixtures of blood containing parasites, their conditioned medium, or exosomes to test this hypothesis. In this experimental setting, we included *Leishmania* exosomes that contain a complex molecular cargo but are also known to carry abundant GP63 (56, 93, 94). Interestingly, our results showed that the GP63 could be detected on the gut’s epithelial layer and muscular fibers during the early phase of the infection. The detection of *Leishmania* GP63 in sand fly vesicular-like structures suggested that GP63 have been internalized in endocytic vesicles; thus, reflecting a potential sand fly gut molecular transport through transcytosis (95). Other metalloproteases can target mammalian epithelial cells. For example, the bacterial metalloproteases gelatinase from *Enterococcus faecalis* (96) and SslE (secreted and surface associated lipoprotein) also known as YghJ from *Escherichia coli* (97) can compromise the integrity of gut epithelial cells in mammals. Therefore, other metalloproteases like GP63 may indeed interact with the host epithelial cells and be internalized by the sand fly gut.

Several studies investigated the cross-talk between intestinal parasites and their hosts, with extracellular vesicles having an important role in this interplay (98, 99). More specifically, the host intestinal cells can internalize exosomes from intestinal worms (100). Nevertheless, the effect of protozoan-secreted molecules or extracellular vesicles on their vectors is scarcely explored.

To test the role of *L. longipapis* SHP-2 during the parasite infection, we used RNAi-mediated gene silencing to suppress its expression. Our results showed that the parasite development was compromised during the early phase of infection, concomitantly with the days when gene silencing was most efficient. Therefore, the suppression of the sand fly immunity repressor potentially caused an increased immune response that caused the reduction of parasites. Interestingly, on an common experimental infection, while parasite multiplication occurs in the early phase of blood digestion, there is an associated increase of gene expression of AMPs attacin, cecropin, and defensin 2 (LlDef2) in *L. longipalpis* (36). In *P. papatasi*, the gene expression of a gut-specific defensin (PpDef1) increased at the end of blood digestion when another parasite multiplicative phase occur (37). Analogously, the suppression of LlDef2 and PpDef1 expression by RNAi-mediated gene silencing favored the parasites (36, 101). These studies corroborate the role of the sand fly immune response in balancing the parasite infection. In addition, to test if the reduction of parasite survival in the earlier infection phase would have a consequent effect on the progress of the infection, we analyzed infection parameters on 8 days post-infection. At this time, it is possible to detect parasites colonizing different parts of the sand fly gut and several differentiation forms, thus reflecting the ability of the parasite to advance in its cycle in the vector. At this stage of infection, the number of infected sand flies, the intensity of parasite loads, and parasite developmental forms did not suffer the impact of the early SHP-2 silencing and parasite reduction. In the context of the parasite GP63 and sand fly SHP-2, our current results and results from Hajmova et al. (88) show that they have an important role in the initial phase of infection. In addition, we observed a non-significant but intriguing increase in parasites localized at the stomodeal valve. This finding reflects that the early suppression of the SHP-2, with a consequent less favorable condition for the parasites, may have caused the variable migration to the stomodeal valve.

In conclusion, our results suggest that, as well as in macrophages, *Leishmania* can activate the invertebrate host equivalent to SHP-1, thus inhibiting the Toll pathway via sand fly immunosuppression. The present work is the first report of a putative *Leishmania* protein capable of modulating the immune response of the insect vector.

## Data availability statement

The datasets presented in this study can be found in online repositories. The names of the repository/repositories and accession number(s) can be found in the article/**Supplementary Material**.

## Author contributions

Conceptualization, YT-C, and ANP. Methodology, ELT, TL, and ANP. Validation, ELT, BT-N, and ANP. Investigation and formal analysis, ELT, BT-N, DMF, TD-B, TL, and ANP. Resources, KPC, YT-C and PV. Data curation, ELT, BT-N, TD-B, and ANP. Writing—original draft preparation, ELT. Review and editing, ELT, KPC, PV, YT-C, and ANP. Visualization, ELT and ANP. Funding acquisition, project administration, and supervision, ELT, PV, YT-C, and ANP. All authors contributed to the article and approved the submitted version.
