# Supplementary material for: Evidence of a conserved mammalian immunosuppression mechanism in Lutzomyia longipalpis upon infection with Leishmania
Source: Front Immunol. 2023 Nov 2;14:1162596. doi: 10.3389/fimmu.2023.1162596 (PMC10652419; doi:10.3389/fimmu.2023.1162596)
Supplement: Supplementary file 1 [file DataSheet_1.pdf]

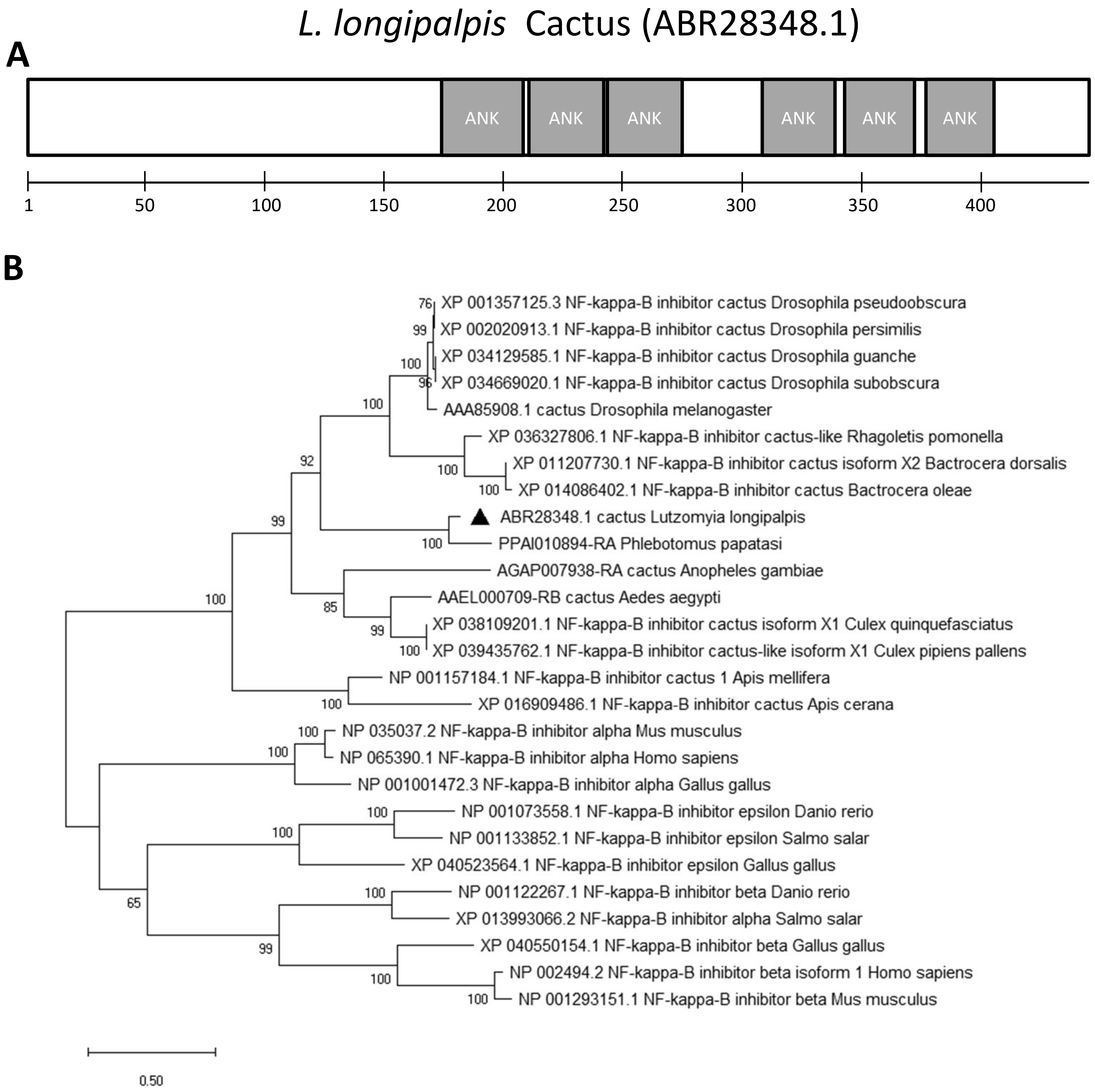

L. longipalpis Cactus (ABR28348.1)
A
ANK
ANK
ANK
ANK
ANK
ANK
1
50
100
150
200
250
300
350
400
B
XP 001357125.3 NF-kappa-B inhibitor cactus Drosophila pseudoobscura
XP 002020913.1 NF-kappa-B inhibitor cactus Drosophila persimilis
XP 034129585.1 NF-kappa-B inhibitor cactus Drosophila guanche
XP 034669020.1 NF-kappa-B inhibitor cactus Drosophila subobscura
AAA85908.1 cactus Drosophila melanogaster
XP 036327806.1 NF-kappa-B inhibitor cactus-like Rhagoletis pomonella
XP 011207730.1 NF-kappa-B inhibitor cactus isoform X2 Bactrocera dorsalis
XP 014086402.1 NF-kappa-B inhibitor cactus Bactrocera oleae
ABR28348.1 cactus Lutzomyia longipalpis
PPAI010894-RA Phlebotomus papatasi
AGAP007938-RA cactus Anopheles gambiae
AAEL000709-RB cactus Aedes aegypti
XP 038109201.1 NF-kappa-B inhibitor cactus isoform X1 Culex quinquefasciatus
XP 039435762.1 NF-kappa-B inhibitor cactus-like isoform X1 Culex pipiens pallens
NP 001157184.1 NF-kappa-B inhibitor cactus 1 Apis mellifera
XP 016909486.1 NF-kappa-B inhibitor cactus Apis cerana
NP 035037.2 NF-kappa-B inhibitor alpha Mus musculus
NP 065390.1 NF-kappa-B inhibitor alpha Homo sapiens
NP 001001472.3 NF-kappa-B inhibitor alpha Gallus gallus
NP 001073558.1 NF-kappa-B inhibitor epsilon Danio rerio
NP 001133852.1 NF-kappa-B inhibitor epsilon Salmo salar
XP 040523564.1 NF-kappa-B inhibitor epsilon Gallus gallus
NP 001122267.1 NF-kappa-B inhibitor beta Danio rerio
XP 013993066.2 NF-kappa-B inhibitor alpha Salmo salar
XP 040550154.1 NF-kappa-B inhibitor beta Gallus gallus
NP 002494.2 NF-kappa-B inhibitor beta isoform 1 Homo sapiens
NP 001293151.1 NF-kappa-B inhibitor beta Mus musculus
76
99
100
96
100
92
100
100
99
100
85
99
100
100
100
100
100
100
100
100
65
99
100
100
0.50

C

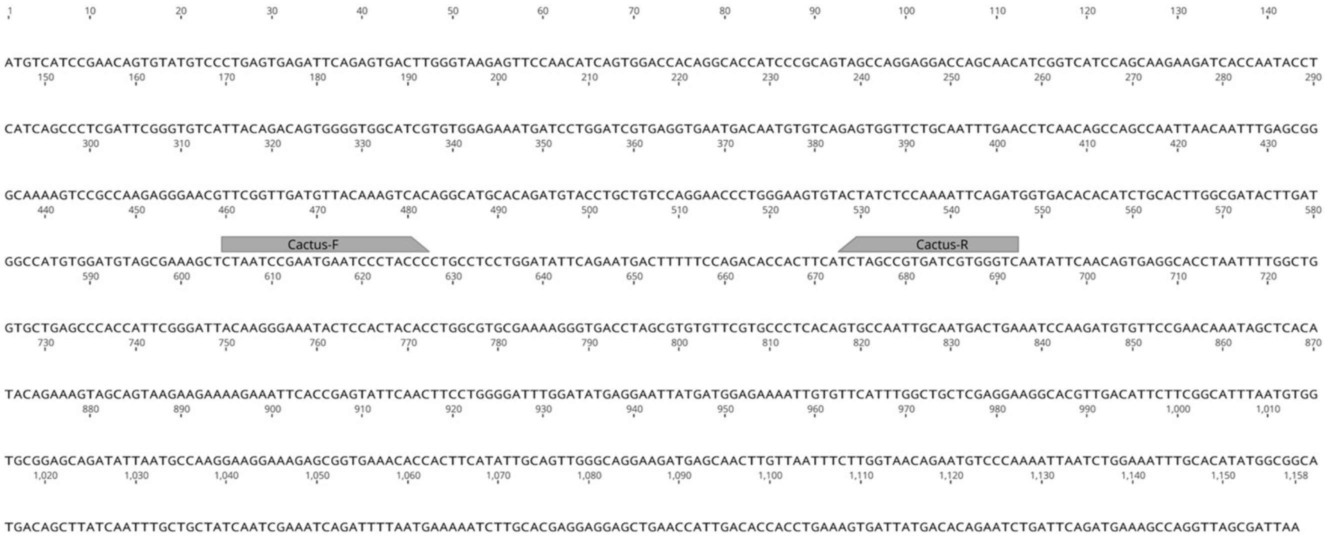

Legend: ***L. longipalpis* cactus sequence. A**- Conserved domains identified on the amino acid sequence (ABR28348.1): grey box indicates the ankyrin (ANK) repeat domains of cactus protein; numeric scale indicates amino acid position. **B**- Phylogram of cactus amino acid sequences from *L. longipalpis* and other organisma inferred by Maximum Likelihood method based on the Jones-Taylor-Thorton model: numbers on branch nodes indicate bootstrap values; Vector Base or GenBank accession numbers are followed by corresponding identification and species names; scale bar indicates number of substitutions per site. **C**- Nucleotide sequence (LLOJ004612-RA) with primer annealing sites indicated in grey.
