# Supplementary material for: Evidence of a conserved mammalian immunosuppression mechanism in Lutzomyia longipalpis upon infection with Leishmania
Source: Front Immunol. 2023 Nov 2;14:1162596. doi: 10.3389/fimmu.2023.1162596 (PMC10652419; doi:10.3389/fimmu.2023.1162596)
Supplement: Supplementary file 2 [file DataSheet_2.pdf]

L. longipalpis SHP-2 (AKU77025.1)

A

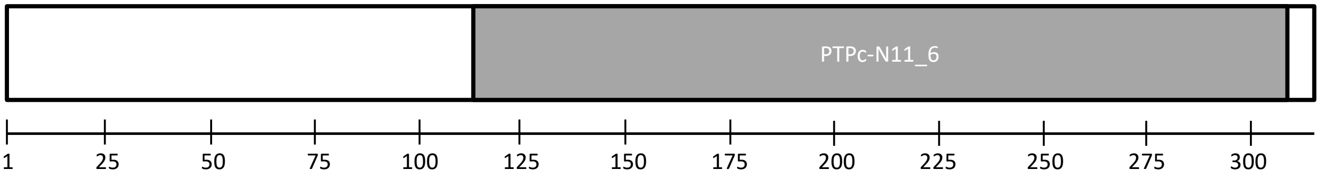

B

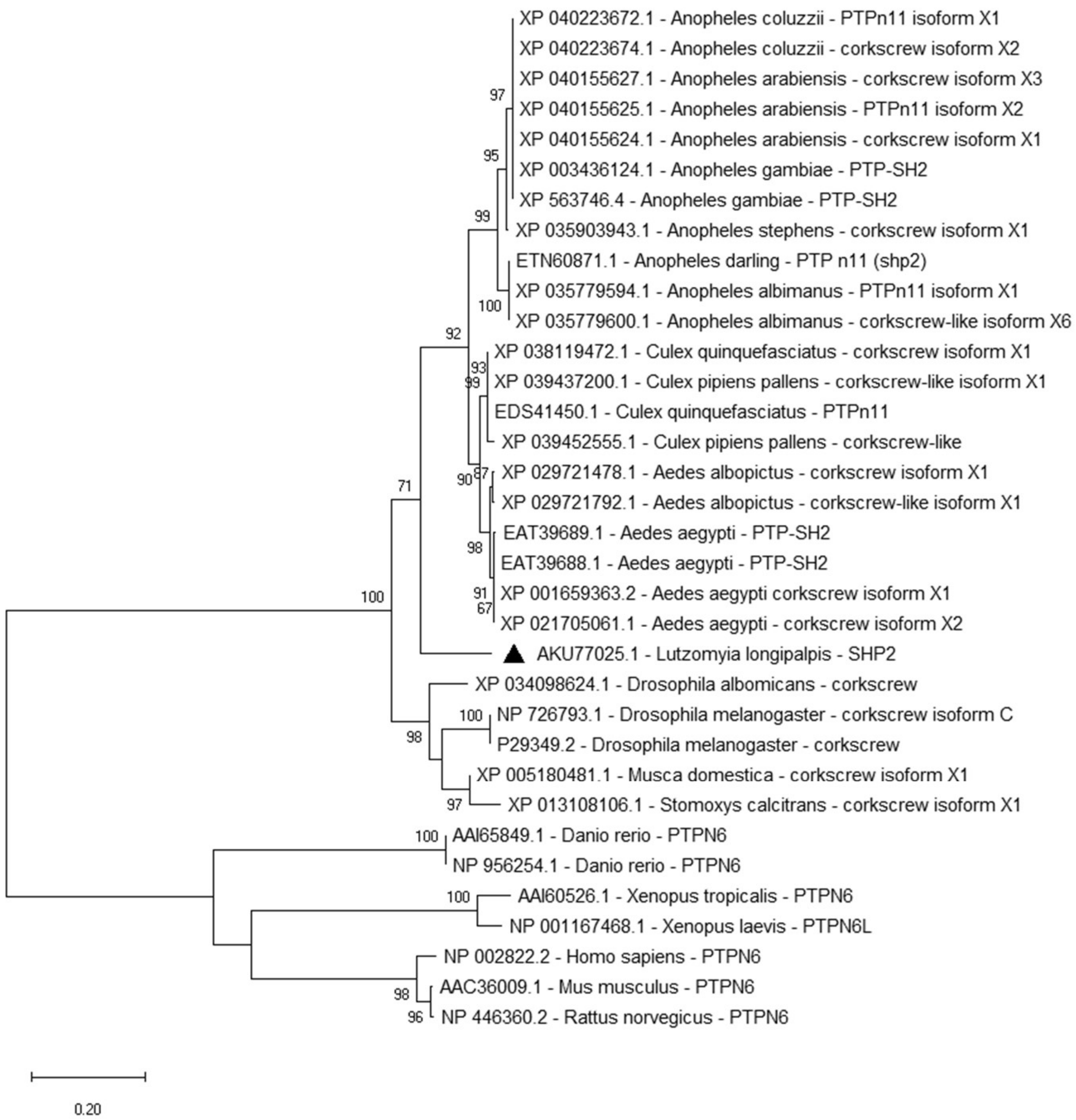

C

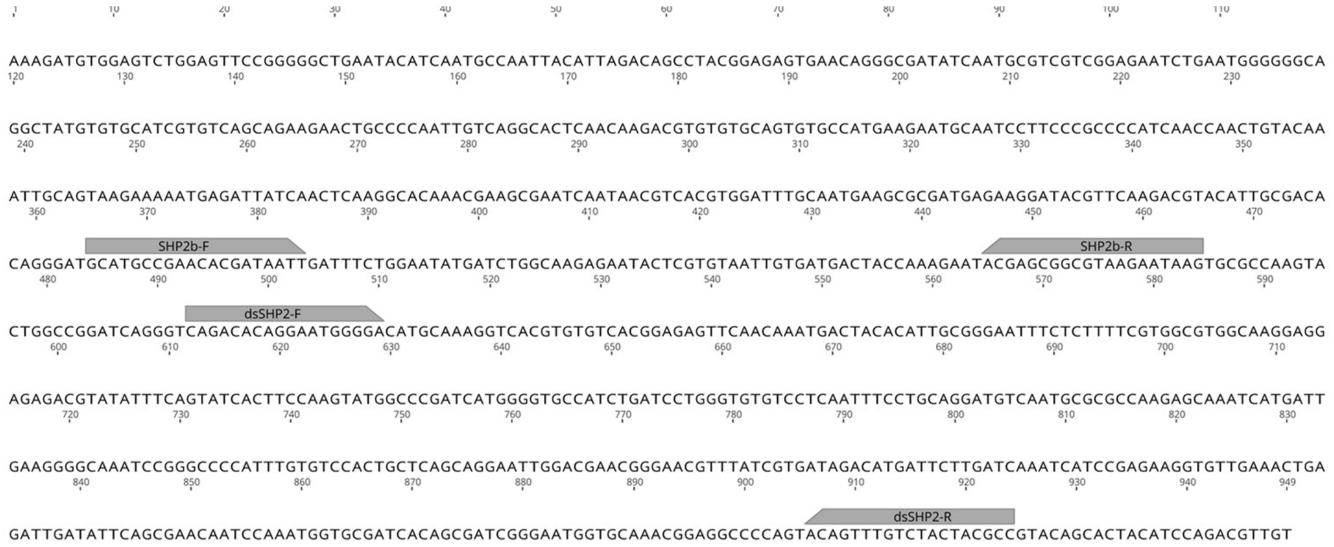

Legend: ***L. longipalpis*** **SHP2 sequence. A**- Signature domains identified on the amino acid sequence (AKU77025.1): grey box indicate the tyrosine-protein phosphatase non-receptor type 11 and type 6 catalytic domain (PTPc-N11_6) (cd14544); numeric scale indicates amino acid position. **B**- Phylogram of PTP amino acid sequences from *L. longipalpis* and other organisms inferred by Maximum Likelihood method based on the Le Gascuel 2008 model: numbers on branch nodes indicate bootstrap values; GenBank accession number are followed by corresponding species names; scale bar indicates number of substitutions per site. **C**- Nucleotide sequence (KP030756.1) with primer annealing sites indicated in grey.
